# *In vivo* knockdown of Piccolino disrupts presynaptic ribbon morphology in mouse photoreceptor synapses

**DOI:** 10.3389/fncel.2014.00259

**Published:** 2014-09-03

**Authors:** Hanna Regus-Leidig, Michaela Fuchs, Martina Löhner, Sarah R. Leist, Sergio Leal-Ortiz, Vince A. Chiodo, William W. Hauswirth, Craig C. Garner, Johann H. Brandstätter

**Affiliations:** ^1^Department of Biology, Animal Physiology, Friedrich-Alexander-University of Erlangen-NurembergErlangen, Germany; ^2^Department of Infection Genetics, Helmholtz Centre for Infection ResearchBraunschweig, Germany; ^3^Department of Psychiatry and Behavioral Sciences, Stanford UniversityPalo Alto, CA, USA; ^4^Department of Ophthalmology, College of Medicine, University of FloridaGainesville, FL, USA; ^5^Deutsches Zentrum für Neurodegenerative ErkrankungenCharité, Berlin, Germany

**Keywords:** retina, ribbon synapse, active zone, Piccolo, Aczonin, Piccolino, RIBEYE

## Abstract

Piccolo is the largest known cytomatrix protein at active zones of chemical synapses. A growing number of studies on conventional chemical synapses assign Piccolo a role in the recruitment and integration of molecules relevant for both endo- and exocytosis of synaptic vesicles, the dynamic assembly of presynaptic F-actin, as well as the proteostasis of presynaptic proteins, yet a direct function in the structural organization of the active zone has not been uncovered in part due to the expression of multiple alternatively spliced isoforms. We recently identified Piccolino, a Piccolo splice variant specifically expressed in sensory ribbon synapses of the eye and ear. Here we down regulated Piccolino *in vivo* via an adeno-associated virus-based RNA interference approach and explored the impact on the presynaptic structure of mouse photoreceptor ribbon synapses. Detailed immunocytochemical light and electron microscopical analysis of Piccolino knockdown in photoreceptors revealed a hitherto undescribed photoreceptor ribbon synaptic phenotype with striking morphological changes of synaptic ribbon ultrastructure.

## Introduction

The active zone (AZ) of chemical synapses is an area at the presynaptic plasma membrane specialized for the release of neurotransmitter. It is composed of the presynaptic plasma membrane and the cytomatrix assembled at the AZ (CAZ). The CAZ includes proteins like Bassoon, Piccolo (also known as Aczonin), RIMs, ELKS/CAST, and Munc13s (Gundelfinger and Fejtová, 2012; Südhof, 2012). Of these, little is known about the synaptic function of Piccolo, the largest multidomain protein of the CAZ (Wang et al., 1999; Fenster et al., 2000). One contributing factor is the lack of knockout mice in which all occurring Piccolo protein variants are deleted (Mukherjee et al., 2010; Waites et al., 2011; Regus-Leidig et al., 2013). The analysis of hippocampal and cortical synapses of mice lacking exon 14 (Pclo^exon14^), which specifically reduces the expression of only the longest 560 kD isoforms, led to the conclusion that Piccolo has little or no function at excitatory synapses (Mukherjee et al., 2010). However, strategies based on RNA interference designed to knockdown all Piccolo isoforms revealed that, while Piccolo is not required for the assembly of CNS synapses nor the localization of other CAZ proteins, its loss leads to an increase in the rates of synaptic vesicle exocytosis and the dispersion kinetics of Synapsin1a (Leal-Ortiz et al., 2008). These phenotypes were subsequently shown to be due to a critical role for Piccolo in the dynamic assembly of presynaptic F-Actin and the activity dependent recruitment of CaMKII and actin modulatory proteins such as Profilin2 (Waites et al., 2011). Importantly, these phenotypes only appear in Pclo^exon14^ mice when all remaining Piccolo isoforms are eliminated (Waites et al., 2011). Intriguingly, other facets of Piccolo function at CNS synapses appear to be masked by compensation of the structurally related CAZ protein Bassoon. Surprisingly, inactivating both proteins causes the loss of synaptic vesicle pools and the disintegration of synaptic junctions. These phenotypes are caused by an enhanced activation of proteins within the proteasome, lysosomal and ubiquitin systems, implying a central role for Piccolo/Bassoon in presynaptic proteostasis (Waites et al., 2011).

Although Piccolo is present at nearly all chemical synapses (Schoch and Gundelfinger, 2006), its function at ribbon synapses of sensory neurons has not been explored. Sensory ribbon synapses are specialized for the fast and tonic release of neurotransmitter and are comprised of a large AZ and an attached ribbon, an electron dense presynaptic organelle tethering a high number of synaptic vesicles (Moser et al., 2006; Matthews and Fuchs, 2010; Regus-Leidig and Brandstätter, 2012). In contrast to conventional synapses, Piccolo and Bassoon do not co-localize at photoreceptor ribbon synapses of the retina. Here, while Bassoon is present at the arciform density/AZ, Piccolo is associated with the synaptic ribbon, suggesting divergent functions for these proteins at this type of sensory synapse (Dick et al., 2001; Limbach et al., 2011). Recently, we have demonstrated that ribbon synapses of the eye and ear express a specific, C-terminally truncated splice variant of Piccolo, which we named Piccolino (Regus-Leidig et al., 2013). The truncation results in the loss of interaction sites for RIMs, ELKS/CAST, Munc13s, and the L-type Ca^2+^ channel, all of which have been shown to exist in the longest 560 kD Piccolo variant (Fujimoto et al., 2002; Shibasaki et al., 2004; Takao-Rikitsu et al., 2004; Wang et al., 2009; Regus-Leidig et al., 2013), perhaps explaining Piccolino's absence from the arciform density at photoreceptor ribbon synapses (Regus-Leidig et al., 2013).

In the current study we examined the impact of Piccolino down regulation by *in vivo* RNA interference in rod photoreceptors of the murine retina. We report that Piccolino down regulation disrupts ribbon morphology, suggesting an involvement of Piccolino in the structural organization of the ribbon.

## Materials and methods

### Ethics statement

The experiments were performed in compliance with the guidelines for the welfare of experimental animals issued by the Federal Government of Germany and the University of Erlangen-Nuremberg. The animal experiments were approved and registered by the local authorities (Regierung von Mittelfranken, AZ 54-2531.31-26/07; Amt für Veterinärwesen der Stadt Erlangen, AZ TS - 10/07 Lehrstuhl für Zoologie-Tierphysiologie). Mouse breeding was performed in the animal facilities of the University of Erlangen-Nuremberg according to European and German laws on experimental animal welfare (Tierschutzgesetz; AZ 820-8791.2.63).

### Animals

In this study C57BL/6 and Balb/c mice of either sex were used. Mice were maintained on a 12/12 h light/dark cycle with light on at 6 am and an average illumination of 200 lux (white light; TLD 58W/25 tubes, Philips, Hamburg, Germany). Light and dark adaptation was performed as follows: mice were light adapted for 3 h or light adapted for 3 h and subsequently dark adapted for 3 h. Dark adapted retinae were prepared and fixed under dim red light.

### rAAV-shRNA vector production and subretinal virus injection

The H1 RNA polymerase III promoter driven Pclo28 cassette from the FUGW H1 vector (Leal-Ortiz et al., 2008; Waites et al., 2013) was inserted into the mOP-GFP-rAAV vector expressing GFP under control of the mouse opsin promotor (Beltran et al., 2010) into the SalI site. The resulting plasmid construct (mOP-GFP-Pclo28-rAAV) was packaged into rAAV5 capsids using standard vector preparation methods (Zolotukhin et al., 1999), and purified and titered as described (Beltran et al., 2010). For subretinal injection of the viral constructs, mouse pups (P5-P7) were anesthetized by hypothermia. The prospective eyelid was numbed by application of a local anesthetic (Xylocain, Astra Zeneca GmbH, Wedel, Germany) and opened with a scalpel. After making a small incision on the nasal side of the sclera, a 34-gauge beveled needle attached to a microliter syringe (#207434 and #7634-01, Hamilton, Bonaduz, Switzerland) was inserted through the hole, and 0.5 μl virus (at a titer between 4 and 6 × 10^12^ vg/ml) was delivered subretinally. Injections were performed on right eyes only, leaving the left eyes as untreated controls. The incision site was treated with an antibiotic ointment (Refobacin, Merck Serono GmbH, Darmstadt, Germany) and pups were warmed up on a heating pad (37°C). The transduction efficiency was observed using a Micron III Retinal Imaging Microscope (Phoenix Research Labs, Pleasanton, CA, USA). Mice were anesthetized by an intramuscular injection of 50 mg/kg ketamine (Ketavet®, Pfizer, Berlin, Germany) and 10 mg/kg xylazine (Rompun® 2%, Bayer, Leverkusen, Germany). A subcutaneous injection of saline solution (10 ml/kg, 0.9%) was administered to prevent desiccation, and pupils were dilated with a drop of tropicamide (Mydriaticum Stulln®, 5 mg/ml, Pharma Stulln, Stulln, Germany) and phenylephrine hydrochloride (Neosynephrin POS® 5%, Ursapharm, Saarbrücken, Germany).

### Cell sorting and quantitative PCR

For sorting of transduced GFP positive photoreceptor cells, injected retinae were dissociated by papain digestion (20 U/ml; Worthington Biochemical, Lakewood, NJ, USA) at 37°C for 20 min with subsequent trituration and resuspension in FACS buffer (2% FCS, 2 mM EDTA in 0.1 M PBS, pH 7.4). Cells were sorted in a MoFlo High Speed Cell Sorter (Beckman Coulter, Krefeld, Germany) at the Nikolaus Fiebiger Center for Molecular Medicine, Erlangen, Germany, and collected in RLT buffer (Qiagen, Hilden, Germany) containing 1% β-Mercaptoethanol. Total RNA was isolated using the RNeasy Micro Kit (Qiagen) and subjected to reverse transcription using the iScript™ cDNA Synthesis Kit (Bio-Rad, Munich, Germany) in a total volume of 20 μl. For quantitative PCR, 1 μl of cDNA was used in a total volume of 12.5 μl containing 6.25 μl of 2× SsoFast EvaGreen Supermix (Bio-Rad) and 160 nM of target gene forward and reverse primer and amplified using an initial denaturation step (3 min at 95°C) followed by 40 cycles (10 s at 95°C; 10 s at 59°C; 10 s at 72°C) using a CFX96 Real-Time PCR Detection System (Bio-Rad). The relative expression of Piccolo isoforms, normalized to *Actb*, *Pbgd*, and *Gapdh*, was calculated using the Bio-Rad CFX Manager 1.6 software (Bio-Rad). The following primer sets were used:

Piccolo: Forward primer: AGCAAAGACAGGACAGAA; Reverse primer: ATATTCCGTCAGAGGAGTAC; ß-actin: Forward primer: ATGCTCCCCGGGCTGTAT; Reverse primer: TCACCCACATAGGAGTCCTTCTG; GAPDH: Forward primer: CAACTTTGTCAAGCTCATT; Reverse primer: TCTGGGATGGAAATTGTG; PBGD: Forward primer: ACAAGATTCTTGATACTGCACTCTCTAAG; Reverse primer: CCTTCAGGGAGTGAACGACCA. Negative controls were treated as above without adding template, and amplicon sizes were verified on 1% agarose gels stained with EtBr (Roth, Karlsruhe, Germany).

### Tissue preparation and light microscopical analysis

Preparation of retinal tissue and antibody incubation for light microscopical immunocytochemistry were done as described (Regus-Leidig et al., 2013; Fuchs et al., 2014). Briefly, the eyes were opened and retinae were immersion fixed in the eyecup for 30 min in 4% paraformaldehyde (PFA) in phosphate buffer (PB; 0.1 M, pH 7.4). The retinae were frozen in freezing medium (Reichert-Jung, Bensheim, Germany), and 16 μm thick vertical sections were cut with a cryostat CM3050 S (Leica, Wetzlar, Germany). Primary antibody incubation was carried out overnight at room temperature, secondary antibody incubation for 1 h. For analysis, labeled sections were examined with a Zeiss Axio Imager Z1 equipped with an ApoTome (Zeiss, Oberkochen, Germany). Images were taken with a 20× (0.8, Apochromat) or 100× (1.3 oil, Plan-Neofluar) objective as stacks of multiple optical sections, and projections were calculated with the AxioVision 4.6.3 software (Zeiss). The images were adjusted for contrast and brightness using Adobe Photoshop CS5 (Adobe, San Jose, CA, USA). 3D reconstructions from z-stacks were generated and analyzed with the Imaris software (Bitplane, Zurich, Switzerland). The threshold for reconstruction of single synaptic ribbons was set automatically by the software.

For whole-mount stainings the eyes were opened and the retinae were immersion-fixed for 15 min in 4% PFA in phosphate buffered saline (PBS; 0.1 M, pH 7.4) followed by increasing sucrose steps for cryo-protection. Before antibody incubation the retinae were dissected out of the eyecup, subjected to four cycles of freezing in liquid nitrogen and thawing at 37°C, and incubated in blocking solution (10% normal goat serum, 1% bovine serum albumin, 0.5% Triton X-100 in PBS) for 60 min. Primary antibody incubation was carried out for 5 days at 4°C and 1 day at room temperature, secondary antibody incubation for 2 h at room temperature. Images were taken with a Zeiss LSM 710 in combination with the Zen 2010 software (Zeiss) with a 63× (1.40 oil, Plan-Apochromat) objective as stacks of multiple optical sections. Images from each antibody staining were acquired using the same camera, laser and photo-multiplier tube settings. The projections of optical sections and length/intensity analyses were done using the Zen 2010 software (Zeiss).

### Electron microscopy

For conventional electron microscopy and good tissue preservation, retinae were fixed in 4% PFA and 2.5% glutaraldehyde for 2 h at room temperature. Tissue contrasting was carried out by incubation in 1.5% potassium ferrocyanide and 2% osmium tetroxide in 0.1 M cacodylate buffer (pH 7.4) for 1.5 h. Retinae were dehydrated using an ethanol series and propylene oxide with 0.5% uranyl acetate added at the 70% ethanol step. The tissue was embedded in Renlam resin (Serva, Heidelberg, Germany). For pre-embedding immunoelectron microscopy, retinae were prefixed in 4% PFA in Soerensen buffer (0.1 M Na_2_HPO_4_·2H_2_O, 0.1 M KH_2_PO_4_, pH 7.4) for 50 min at room temperature and further processed as described previously (Regus-Leidig et al., 2013; Fuchs et al., 2014). Briefly, after four cycles of freezing in liquid nitrogen and thawing at 37°C, retinae were PBS washed and embedded in buffered 2% Agar. Agar blocks were sectioned into 100 μm sections with a vibratome VT 1000 S (Leica). The sections were incubated in 10% normal goat serum, 1% bovine serum albumin in PBS for 2 h, followed by incubation with primary antibodies for 4 days at 4°C. PBS washed sections were incubated with biotinylated secondary antibodies (1:500), and visualized by Vectastain ABC-Kit (both from Vector Laboratories, Burlingame, CA, USA). The sections were fixed in 2.5% glutaraldehyde in cacodylate buffer. Diaminobenzidine precipitates were silver enhanced and postfixed in 0.5% OsO_4_ in cacodylate buffer for 30 min at 4°C. Dehydrated specimens were flat-mounted between ACLAR®-films (Ted Pella Inc., Redding, CA, USA) in Epon resin (Fluka, Buchs, Switzerland).

Ultrathin sections were stained with uranyl acetate and lead citrate. The sections were examined and photographed with a Zeiss EM10 electron microscope (Zeiss) and a Gatan SC1000 OriusTM CCD camera (GATAN, Munich, Germany) in combination with the DigitalMicrograph™ 3.1 software (GATAN, Pleasanton, CA, USA). Images were adjusted for contrast and brightness using Adobe Photoshop CS5 (Adobe). 3D reconstructions from serial sections were created with Reconstruct v1.1.0.0 (J. Fiala, Medical College of Georgia). The heights of plate-shaped ribbons and diameters of spherical ribbons were measured using Image J (Schneider et al., 2012).

### Antibodies

The following primary antibodies were used for pre-embedding immunoelectron microscopy (pre-EM), immunocytochemistry on cryostat sections (ICC) and whole-mount retinae (WM): Monoclonal mouse anti-Bassoon mab7f (ICC 1:2500; #VAM-PS003E Stressgen, North Yorkshire, UK), mouse anti-Piccolo (WM 1:5000; #142111 Synaptic Systems, Göttingen, Germany), polyclonal rabbit anti-Bassoon 1.6 (WM 1:2500; kind gift of E.D. Gundelfinger, Leibniz Institute for Neurobiology, Magdeburg, Germany), rabbit anti-GFP (ICC 1:2000; pre-EM 1:100000; #A11122 Life Technologies, Carlsbad, CA, USA), rabbit anti-ubMunc13-2 (ICC 1:6000; WM 1:3000; Cooper et al., 2012), rabbit anti-RIBEYE A-domain (ICC 1:50000; WM 1:2500; #192103 Synaptic Systems), guinea-pig anti-GFP (WM 1:200; this antibody was generated and affinity-purified as described in Mühlhans et al. (2011); peptide immunization of guinea pigs was performed by Pineda Antikörper-Service, Berlin, Germany), and guinea pig anti-Piccolo 44a (ICC 1:4000; Dick et al., 2001). The following secondary antibodies were used for ICC and WM: Alexa™ 488 and Alexa™ 568 goat anti-mouse, goat anti-rabbit, and goat anti-guinea pig IgG conjugates (1:500; Molecular Probes, Eugene, OR, USA), Cy3 (1:200) and Cy5 (1:100) goat anti-mouse, and goat anti-rabbit IgG conjugates (Dianova, Hamburg, Germany).

## Results

### *In vivo* transduction efficiency of AAV5-delivered Piccolo shRNA in mouse retina

**Figure 1:**
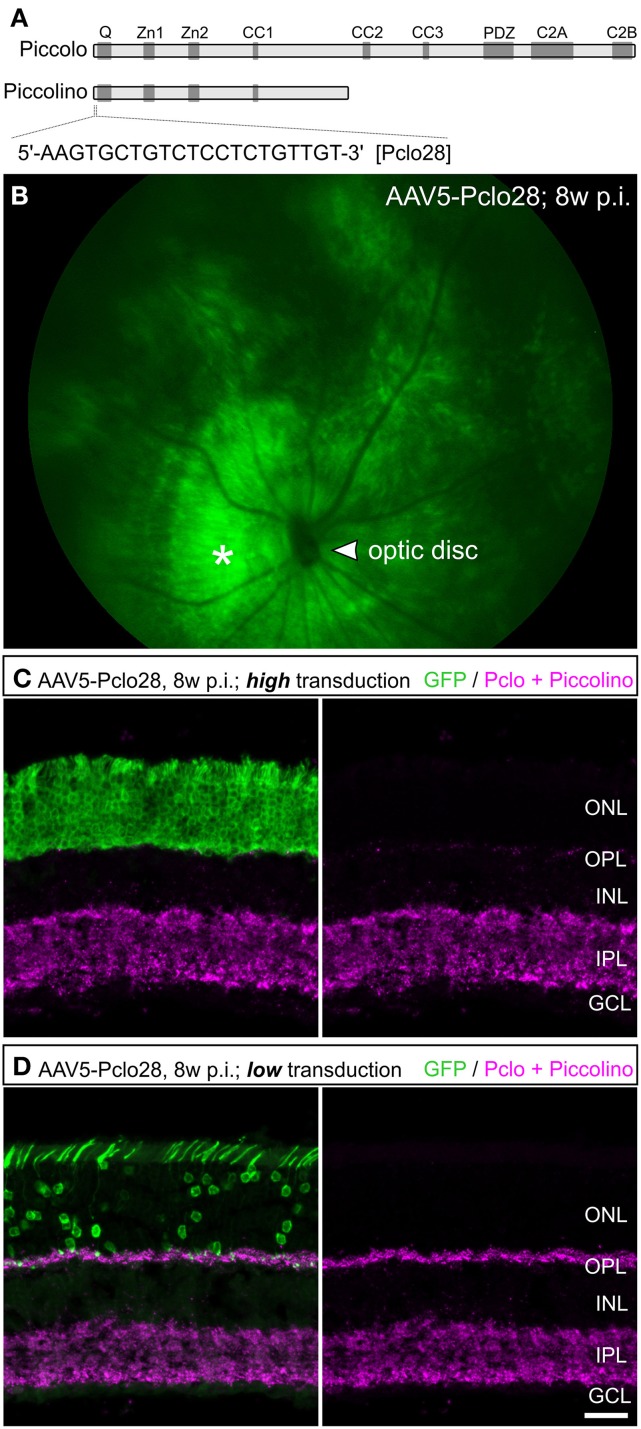
**Targeted delivery of Piccolo shRNA to rod photoreceptors of the mouse retina via subretinal injection of AAV5. (A)** Schematic multidomain structure of the conventional and ribbon synaptic Piccolo variants (Piccolo and Piccolino, respectively), and location and sequence of the shRNA (Pclo28) used to down regulate Piccolino. **(B)** *In vivo* fluorescence fundus photography showing mouse opsin promoter driven GFP expression in transduced rod photoreceptors 8 weeks post injection (8w p.i.). The asterisk demarcates the approximate site of injection where the transduction rate is highest. **(C,D)** Images of vertical sections through an AAV5-Pclo28-transduced retina displaying high **(C)** and low **(D)** transduction rate, stained with an antibody against GFP (green) and the antibody Pclo44a, labeling both Piccolo and Piccolino (magenta). ONL: outer nuclear layer; OPL: outer plexiform layer; INL: inner nuclear layer; IPL: inner plexiform layer; GCL: ganglion cell layer. Scale bar in **(D)** for **(C,D)**: 20 μm.

For Piccolino down regulation in rod photoreceptors, we used an shRNA, which was previously shown to successfully down regulate all Piccolo isoforms in cultured hippocampal neurons when introduced via lentiviral transduction (Pclo28; Leal-Ortiz et al., 2008; Waites et al., 2011). The target sequence for Pclo28 is located in Exon 1 of Piccolo, which encodes segments of the full-length Piccolo as well as the C-terminally truncated, ribbon synapse specific Piccolino (Figure 1A; Fenster and Garner, 2002; Regus-Leidig et al., 2013). To interfere with Piccolo/Piccolino expression early during mouse photoreceptor development and to obtain stable long-term gene silencing, we delivered Pclo28 by means of the rAAV system (see Materials and Methods). The viral particles were injected into the subretinal space between postnatal day 5 (P5) and P7. We also tried injecting P0 mice, which resulted in extensive retinal damage and was therefore not pursued. Virally transduced rod photoreceptors were identified by GFP fluorescence driven by the mouse opsin promoter. GFP expression was detectable 2 weeks post injection (2w p.i.; earliest time point examined) and could be seen up to 6 months p.i. (latest time point examined). Figure 1B shows an example of an *in vivo* fluorescence fundus photography from an AAV5-mOP-GFP-Pclo28 (AAV5-Pclo28) transduced retina 8w p.i. Rod photoreceptor transduction rates (GFP; green) were highest in the immediate vicinity of the injection site (Figure 1B; asterisk), and declined with increasing distance from the injection site.

### *In vivo* knockdown efficiency of AAV5-delivered Piccolo shRNA in mouse rod photoreceptor cells – areas with high vs. low transduction rates

To assess the Pclo28 knockdown efficiency, we first FACS-sorted GFP-positive photoreceptors from dissociated AAV5-Pclo28-transduced retinae 8w p.i. and compared the amount of Piccolo mRNA with that from isolated AAV5-mOP-GFP (AAV5-GFP; control) transduced photoreceptors by quantitative PCR. Normalized Piccolo mRNA levels in AAV5-Pclo28-transduced rod photoreceptors were reduced to 58 ± 10% of the control levels (*n* = 8 retinae each for AAV5-Pclo28 and AAV5-GFP). The standard deviation probably reflects the variability in knockdown efficiency due to the varying degrees of transduction efficiency and hence varying numbers of viral particles per cell.

Next we explored whether AAV5-delivered Pclo28 also reduced synaptic Piccolino protein in transduced rod photoreceptors. For this we immunostained vertical cryostat sections of transduced retinae 8w p.i. with an antibody against GFP and the antibody Pclo44a (magenta; Figures 1C,D). Note, this antibody not only labels full-length Piccolo but also the shorter, ribbon synapse specific Piccolino (Regus-Leidig et al., 2013). In retinal areas with high transduction rates we observed a dramatic loss of Piccolino immunoreactivity in the outer plexiform layer (OPL; Figure 1C), consistent with the loss of Piccolino from photoreceptor ribbon synapses. Due to the high photoreceptor tropism of AAV5, Piccolo/Piccolino-levels in the inner plexiform layer (IPL) of highly transduced retinal areas appeared unaffected (Figure 1C). In retinal areas with lower transduction rates, Piccolino staining was still detectable in the OPL due to the low number of transduced photoreceptor terminals which are surrounded by many non-transduced photoreceptor terminals (Figure 1D). To exclude side effects of the viral transduction or GFP expression, AAV5-GFP-transduced rod photoreceptor terminals were analyzed as a control. There was no difference in Piccolino staining between transduced and non-transduced retinal areas (data not shown).

**Figure 2:**
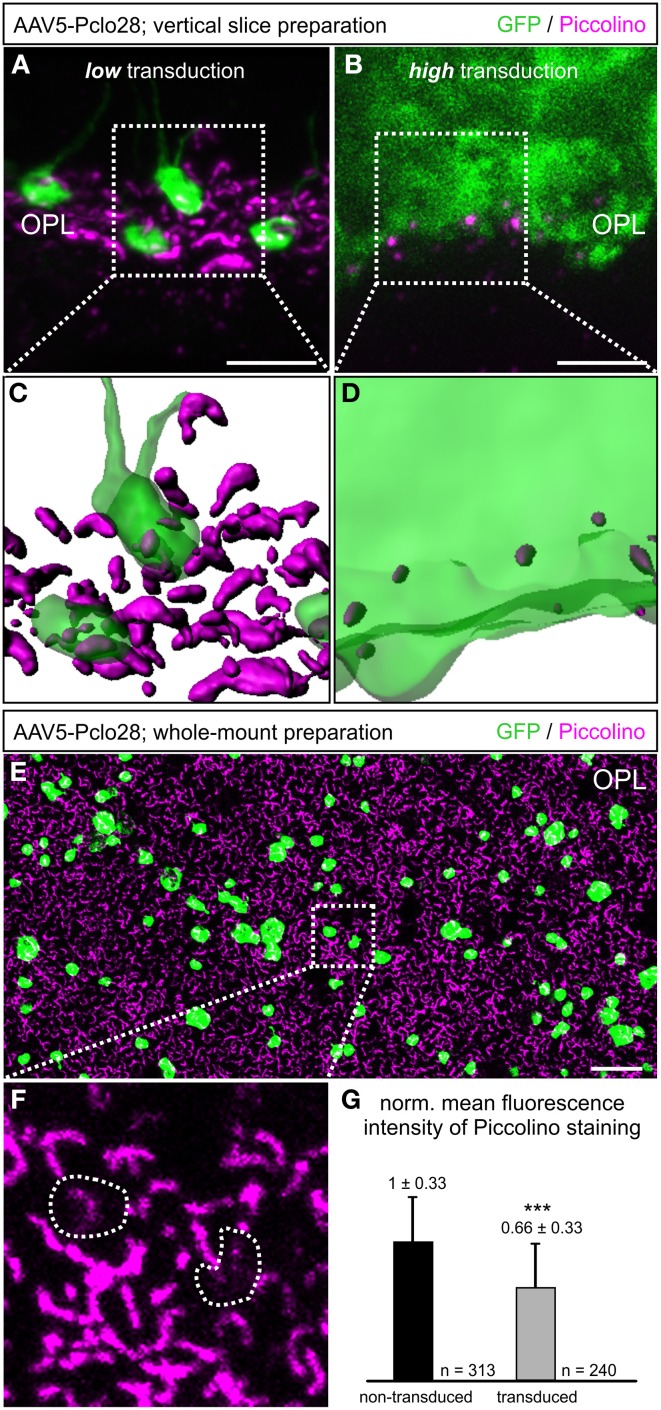
**Piccolino knockdown efficiency in rod photoreceptor terminals. (A,B)** Staining of GFP (green) and Piccolino (magenta; antibody Pclo44a) in vertical cryostat sections through retinal areas with low **(A)** and high **(B)** AAV5-Pclo28-transduction rates 8w p.i. **(C,D)** 3D reconstructions of the GFP and Piccolino staining of the boxed regions in **(A)** and **(B)**. **(E)** Staining of GFP (green) and Piccolino (magenta; antibody Pclo44a) in an AAV5-Pclo28-transduced whole-mounted retina 8w p.i. Projection of a confocal image stack through the outer plexiform layer (OPL). **(F)** Higher magnification of the boxed region in **(E)**. GFP positive rod spherules are outlined with dotted lines. **(G)** Quantification of Piccolino fluorescence staining intensity in non-transduced vs. AAV5-Pclo28-transduced rod photoreceptor terminals. Bars show the mean fluorescence intensity ± SD normalized to non-transduced terminals (^***^p < 0.001; *t*-test). Scale bar in **(A,B)**: 5 μm, in **(E)**: 10 μm.

For better comparison of Pclo28 knockdown efficiency between retinal areas with low and high transduction rates, we evaluated Piccolino staining in vertical cryostat sections 8w p.i. in greater detail (Figures 2A–D). Piccolino staining in transduced rod photoreceptor terminals from retinal areas with low transduction rates sometimes lost its typical horseshoe shape which can be seen in adjacent non-transduced rod photoreceptor terminals (magenta; Figure 2A). The effect of the Pclo28 knockdown on the Piccolino staining was most obvious in photoreceptor terminals from retinal areas with high transduction rates. Here, Piccolino staining was rarely horseshoe-shaped and appeared mostly as small fluorescent puncta (Figure 2B).

The appearance of Piccolino staining in transduced photoreceptors can be visualized best in the 3D-reconstructed z-stacks of the projections displayed in Figures 2A,B shown in Figures 2C,D. Assuming a comparable overall number of rod photoreceptor terminals in both reconstructed retinal areas, it becomes apparent that the Piccolino knockdown is highest, and Piccolino almost absent from most rod photoreceptor synaptic sites in retinal areas with high transduction rates. The difference in Piccolino knockdown efficiency between retinal areas with high and low transduction rates most likely correlates with the number of viral particles per photoreceptor cell, but was not analyzed in greater detail.

As retinae with moderate to low transduction rate/Piccolino knockdown efficiency were more frequently obtained with our subretinal injection approach, we first focused our analyses on these retinal areas. To obtain a higher number of single transduced rod photoreceptor terminals for evaluation, we turned from vertical sections to whole-mount retinal preparations and performed horizontal imaging of the OPL (Figures 2E–G). In the transduced rod photoreceptor terminals, Piccolino staining often displayed the typical horseshoe shape, but sometimes it appeared diffuse or discontinuous (Figure 2F; *dotted lines*). The mean fluorescence intensity of the Piccolino staining in transduced terminals was significantly reduced by one third (*p* < 0.001, *t*-test; Figure 2G), correlating well with the reduction in Piccolo mRNA levels in isolated rod photoreceptors from retinal areas with low transduction rate.

### Light and electron microscopical analysis of the presynaptic ribbon complex in rod photoreceptor terminals from retinal areas with low AAV-Pclo28 transduction rates

**Figure 3:**
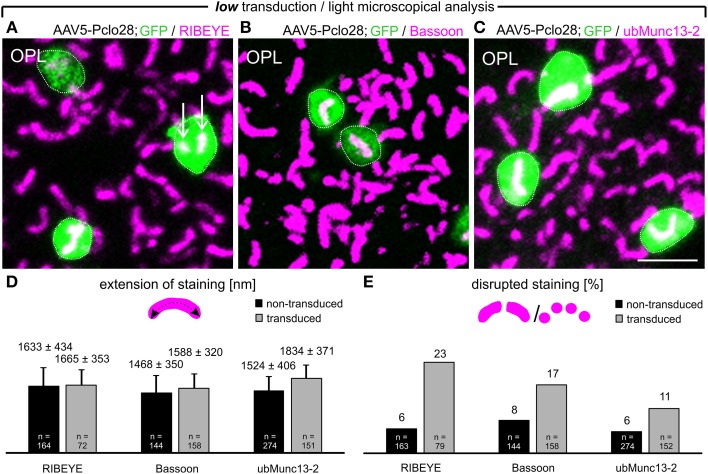
**Whole-mount preparations of AAV5-Pclo28-transduced retinae and analysis of the Piccolino-knockdown effect on RIBEYE, Bassoon, and ubMunc13-2 in rod photoreceptor terminals. (A-C)** Double labeling of GFP (green) with RIBEYE (magenta; **A**), Bassoon (magenta; **B**), and ubMunc13-2 (magenta; **C**) in AAV5-Pclo28-transduced whole-mounted retinae 8w p.i. Projections of confocal image stacks from the OPL. Arrows in **(A)** point to irregular RIBEYE staining. GFP positive rod spherules are outlined with dotted lines. **(D)** Quantification of the lateral extension (= active zone length) of RIBEYE, Bassoon, and ubMunc13-2 staining in non-transduced vs. AAV5-Pclo28-transduced rod photoreceptor terminals. Bars show the mean extension in nm (± SD). **(E)** Percentage of disrupted or punctate RIBEYE, Bassoon, and ubMunc13-2 staining in non-transduced vs. AAV5-Pclo28-transduced rod photoreceptor terminals. OPL: outer plexiform layer. Scale bar in **(C)** for **(A–C)**: 2 μm.

Next we analyzed with light and electron microscopy the impact of the moderate Piccolino knockdown on the structure of the presynaptic ribbon complex. For the immunocytochemical analysis, we chose representative proteins for the different compartments of the presynaptic ribbon complex (tom Dieck et al., 2005): RIBEYE for the ribbon (Schmitz et al., 2000), and Bassoon (Dick et al., 2003) and ubMunc13-2 (Cooper et al., 2012) for the arciform density/AZ compartment. We stained whole-mount preparations of lowly AAV5-Pclo28-transduced retinae 8w p.i. with antibodies against GFP and against the respective protein (Figures 3A–C). For each protein, we determined the two-dimensional extension of the stainings (Figure 3D), and the percentage of irregular horseshoe shapes (disrupted or punctate staining; Figure 3E) in transduced vs. neighboring non-transduced rod photoreceptor terminals. There was little difference in the extension of the stainings for RIBEYE and Bassoon between AAV5-Pclo28-transduced and non-transduced rod photoreceptor terminals, and the staining for ubMunc13-2 was only slightly increased in the transduced terminals (Figure 3D). For the extension of stainings, only contiguous stainings were measured. We are aware that length summation of individual, disrupted ribbon pieces might have revealed a slight difference in AZ length between transduced and non-transduced terminals, but such measurements were technically difficult and therefore not done. For all examined proteins, however, we found a higher percentage of disrupted stainings in AAV5-Pclo28-transduced vs. non-transduced rod photoreceptor terminals, which was most obvious for the RIBEYE staining (Figure 3E). Since mouse rod photoreceptor terminals usually contain only one large AZ with a single synaptic ribbon, two or more fluorescent puncta per terminal which, if connected, would adopt a horseshoe shape were considered as a disrupted ribbon or AZ structure.

**Figure 4:**
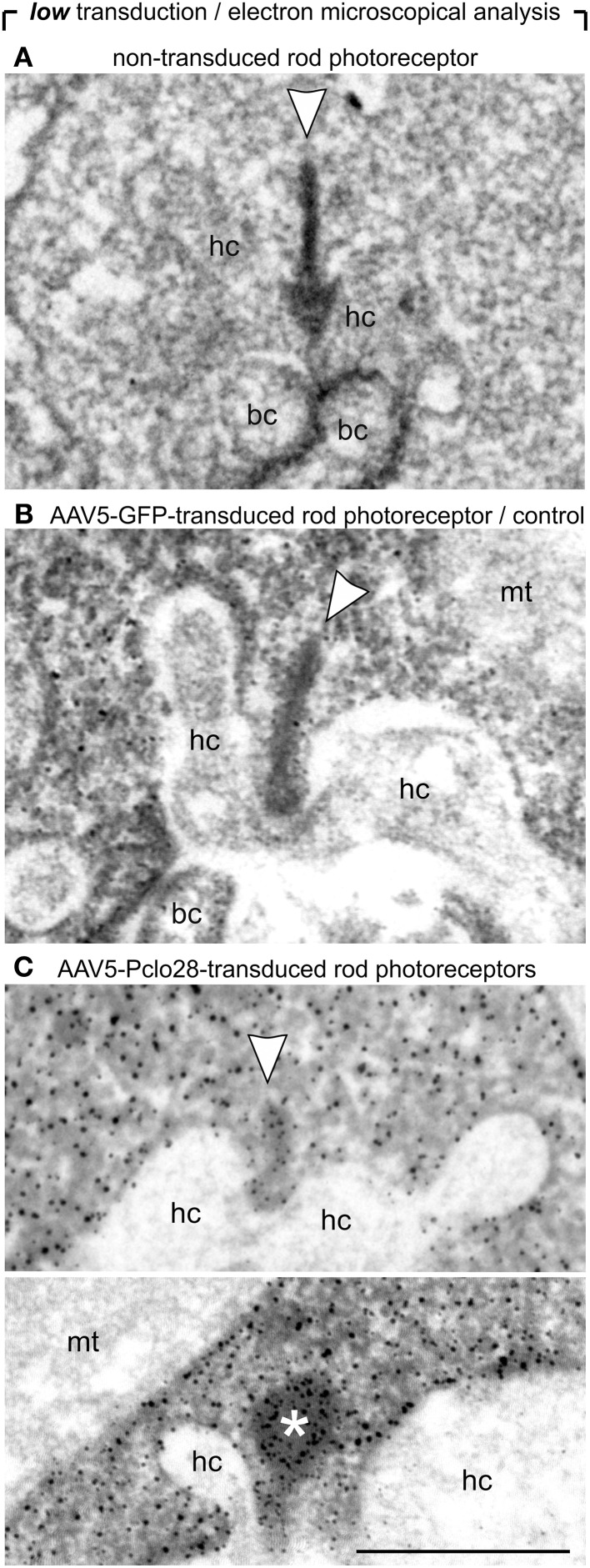
**Ultrastructural analysis of C57BL/6 rod photoreceptor ribbon synapses in retinal areas with low AAV5-Pclo28-transduction rates. (A–C)** Representative electron micrographs of non-transduced **(A)**, AAV5-GFP-transduced (**B**; control), and AAV5-Pclo28-transduced **(C)** rod photoreceptor terminals from retinal areas with low transduction rate. Pre-embedding immunolabeling of GFP in the transduced photoreceptor terminals **(B,C)**. Arrowheads point to synaptic ribbons, the asterisk demarcates spherical, membrane-associated ribbon material. bc: bipolar cell; hc: horizontal cell; mt: mitochondrion. Scale bar in **(C)** for **(A–C)**: 0.5 μm.

Next we analyzed the impact of the moderate Piccolino knockdown on the ultrastructure of the presynaptic ribbon complex in rod photoreceptor terminals (Figure 4). To distinguish transduced from non-transduced rod photoreceptor terminals, we performed pre-embedding immunolabeling of vibratome slices from retinal areas with low AAV5-Pclo28-transduction rates with an antibody against GFP. The terminals of the transduced rod photoreceptors could be easily recognized by the electron-dense GFP staining in the cytoplasm (Figures 4B,C). Ribbon ultrastructure was analyzed in an orthogonal plane of section through the ribbon synaptic complex, defined by the presynaptic ribbon as the central element opposed by two postsynaptic horizontal cell processes. In terminals of non-transduced rod photoreceptors (Figure 4A) and of AAV5-GFP-transduced rod photoreceptors from control retinae (Figure 4B), the synaptic ribbon displayed its typical plate-like shape, extending perpendicular to the presynaptic membrane into the cytoplasm. In many AAV5-Pclo28-transduced rod photoreceptor terminals, however, synaptic ribbons appeared reduced in height (Figure 4C; upper micrograph), swollen or spherically shaped (Figure 4C; lower micrograph). Because of the poor tissue preservation resulting from the fixation and staining procedure, the exact shape of the ribbons was difficult to analyze in detail. For quantification, we therefore divided the rod photoreceptor synaptic ribbon shapes into only two categories: plate-shaped and swollen. The quantitative analysis revealed that the percentage of normal plate-shaped ribbons was 80% in the AAV5-Pclo28-transduced rod photoreceptors while it was around 94% in both non-transduced and AAV5-GFP-transduced rod photoreceptors (AAV5-Pclo28-transduced: 80.0%, *n* = 99 synaptic ribbons from 3 animals; non-transduced: 94.7%, *n* = 252 synaptic ribbons from 3 animals; AAV-GFP-transduced: 93.5%, *n* = 75 synaptic ribbons from 3 animals).

### Light and electron microscopical analysis of the presynaptic ribbon complex in rod photoreceptor terminals from retinal areas with high AAV-Pclo28 transduction rates

**Figure 5:**
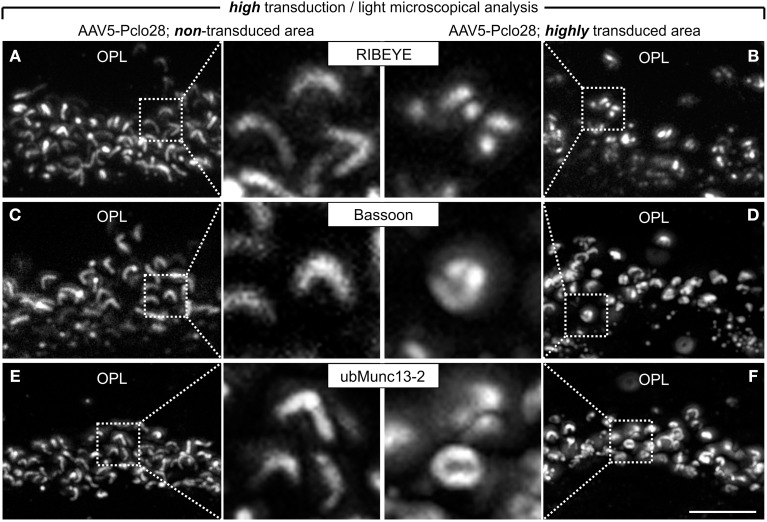
**Vertical sections through retinal areas with high AAV5-Pclo28-transduction rates and analysis of the Piccolino-knockdown effect on RIBEYE, Bassoon, and ubMunc13-2 in rod photoreceptor terminals. (A–F)** Staining for RIBEYE **(A,B)**, Bassoon **(C,D)**, and ubMunc13-2 **(E,F)** in AAV5-Pclo28-transduced retina 8w p.i. For a given staining, images were taken from a non-transduced **(A,C,E)** and a highly transduced area **(B,D,F)** of the same retinal slice. GFP fluorescence is not shown. OPL: outer plexiform layer. Scale bar in **(F)** for **(A–F)**: 5 μm.

The analysis of retinal areas with low transduction rates indicated that a moderate reduction in Piccolino-levels results in ribbon swelling or even in the formation of spherical ribbon material. Since Piccolino was almost undetectable immunocytochemically in highly AAV5-Pclo28-transduced retinal areas (Figures 1, 2), we expected that rod photoreceptor ribbon synaptic sites of such areas would display a more stable and distinct phenotype. Therefore, we performed stainings with antibodies against GFP in combination with antibodies against RIBEYE, Bassoon, or ubMunc13-2, on vertical sections from retinae 8w p.i containing both non-transduced as well as highly AAV5-Pclo28 transduced areas (Figures 5A–F). In rod photoreceptor terminals from non-transduced retinal areas, the stainings for RIBEYE, Bassoon, and ubMunc13-2 showed the typical horseshoe shape of the presynaptic ribbon complex (Figures 5A,C,E). In contrast, RIBEYE staining in highly transduced areas was rarely horseshoe-shaped and appeared mostly as small fluorescent puncta (Figure 5B), concurring with the sometimes observed swollen or spherical ribbon material in the electron microscopical analysis of rod photoreceptor ribbon synapses from retinal areas with low transduction rates (Figure 4C). The stainings for Bassoon and ubMunc13-2 in rod photoreceptor terminals from highly AAV5-Pclo28-transduced areas were also altered and often appeared irregular and sometimes circular instead of horseshoe-shaped (Figures 5D,F).

**Figure 6:**
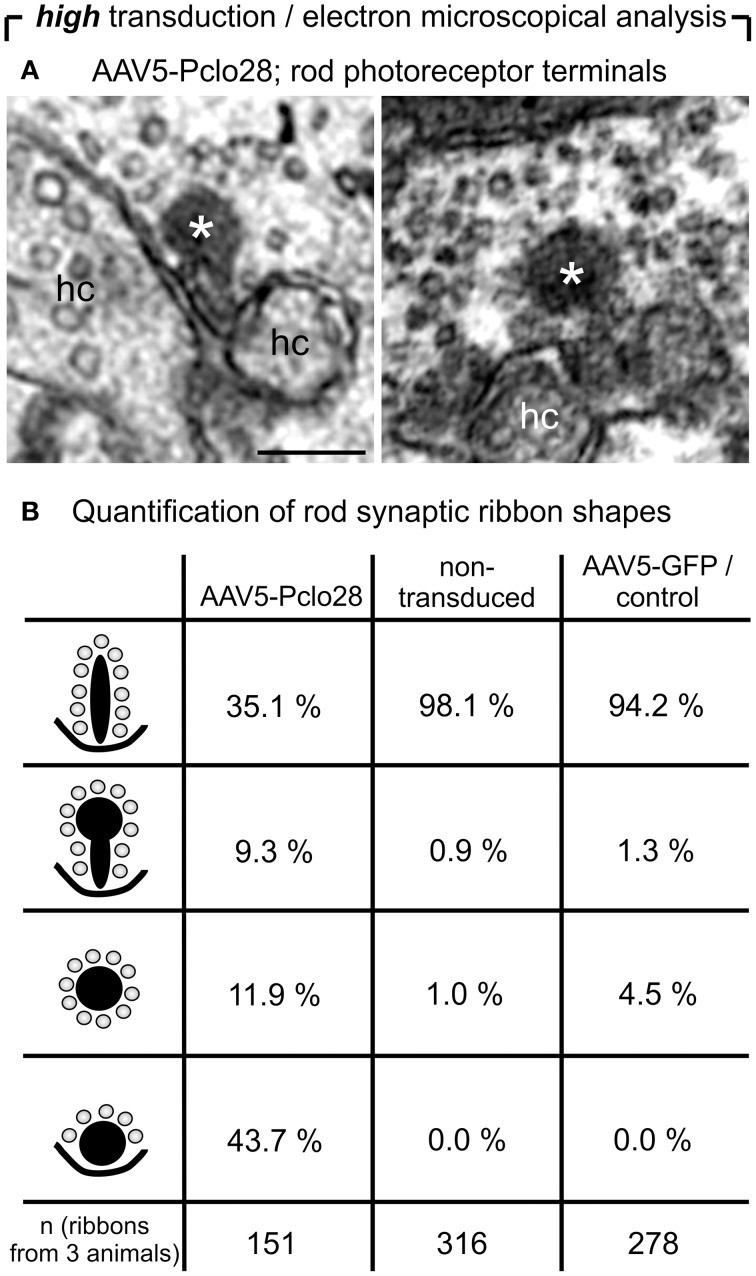
**Ultrastructural analysis of C57BL/6 rod photoreceptor ribbon synapses in retinal areas with high AAV5-Pclo28-transduction rates. (A)** Representative electron micrographs of rod photoreceptor terminals from a highly AAV5-Pclo28-transduced retinal area, processed for best tissue preservation. The asterisks demarcate spherical, membrane-associated ribbon material. **(B)** Quantification of four different shapes of ribbon profiles—plate-shaped, club-shaped, floating and membrane-attached spheres—in rod photoreceptor terminals from highly AAV5-Pclo28-transduced, non-transduced, and AAV5-GFP-transduced (control) retinal areas. hc: horizontal cell. Scale bar in **(A)**: 0.2 μm.

For the ultrastructural analysis of highly AAV5-Pclo28-transduced retinal areas, we analyzed retinal material which was not pre-embedding immunolabeled with an antibody against GFP, but processed for best tissue preservation. The material was excised from retinal areas with high transduction rates showing strong homogenous green fluorescence, assuming that the small number of non-transduced rod photoreceptor terminals within such areas was negligible for the evaluation. Non-transduced and AAV5-GFP-transduced areas were used as controls. As expected, spherical membrane-bound ribbon material was often detectable in rod photoreceptor terminals; two examples of this phenotype in a highly AAV5-Pclo28-transduced retinal area are shown in Figure 6A. The optimal tissue preservation further enabled us to analyze the ribbon phenotype in greater detail. We therefore divided the ribbon shapes for quantification into four categories: plate-shaped, club-shaped, spherical (free floating), and spherical (membrane-attached) (Figure 6B). While over 90% of ribbons in non-transduced and AAV5-GFP-transduced rod photoreceptor terminals were plate-shaped, their percentage decreased to 35% in terminals of AAV5-Pclo28-transduced rod photoreceptors. The remaining 65% of ribbons were club-shaped, spherical free floating, and spherical attached (Figure 6B). Furthermore, the mean height of the remaining plate-shaped synaptic ribbons in AAV5-Pclo28-transduced rod photoreceptor terminals was reduced by ~50% (AAV5-Pclo28-transduced: 118 nm ± 61 nm, SD, *n* = 104 ribbons; non-transduced: 249 nm ± 46 nm, SD, *n* = 121 ribbons; AAV5-GFP-transduced: 240 nm ± 65 nm, SD, *n* = 153 ribbons). The attached spherical ribbons spheres in AAV5-Pclo28-transduced rod photoreceptor terminals had an average diameter of 129 nm ± 27 nm (SD; *n* = 92 ribbon spheres from 3 animals) (Table 1).

**Table 1:** **Mean diameter of spherical ribbon profiles**.

**C57BL/6**			**BALB/c**		
	**P4–P14 wild-type (Regus-Leidig et al., 2009)**	**Adult AAV5-Pclo28 (this study)**	**Adult light-adapted non-transduced (this study)**	**Adult light-adapted AAV5-Pclo28 (this study)**	**Adult dark-adapted AAV5-Pclo28 (this study)**
Free Floating	129 ± 36 nm	n.a.	123 ± 33 nm	124 ± 43 nm	n.a
Membrane-Attached	n.a.	129 ± 27 nm	n.a	131 ± 40 nm	122 ± 37 nm
*n* (spheres)	143	92	154	83	70

### Lack of dynamic ribbon assembly in AAV5-Pclo28-transduced rod photoreceptors from BALB/c mouse retina

The occurrence of spherical ribbon material in photoreceptor terminals is a sign of pathological, turnover or developmental processes. Synaptic ribbon spheres are commonly found at malfunctioning photoreceptor ribbon synapses as a consequence of the disintegration of the plate-shaped ribbon (Dick et al., 2003; Haeseleer et al., 2004; Grossman et al., 2009; Reim et al., 2009; Regus-Leidig et al., 2010b; Fuchs et al., 2012), and spherical ribbon material pinching off of photoreceptor ribbons has been described for albinotic BALB/c mice. Interestingly, the process of ribbon disassembly in BALB/c mice is dynamic and reversible. Light triggers the disassembly process and darkness reverses it, leading to a full recovery of plate-shaped photoreceptor ribbons (Adly et al., 1999; Spiwoks-Becker et al., 2004; Fuchs et al., 2013). Finally, precursor spheres, spherical ribbon material occurring during photoreceptor synaptogenesis, are transport units for CAZ proteins like RIBEYE, Bassoon, and Piccolino in the formation of photoreceptor ribbon synapses (Regus-Leidig et al., 2009).

**Figure 7:**
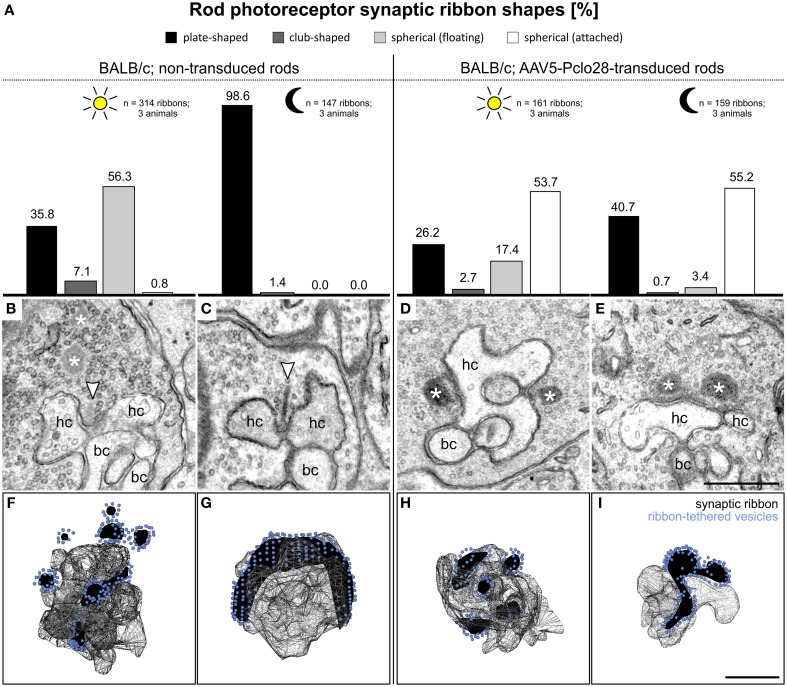
**Ultrastructural analysis of rod photoreceptor ribbon synapses in the AAV5-Pclo28-transduced BALB/c retina. (A)** Percentages of the four different shapes of ribbon profiles—plate-shaped, club-shaped, floating and membrane-attached spheres—in non-transduced and AAV5-Pclo28-transduced rod photoreceptor terminals of BALB/c mice after 3 h light and dark adaptation. **(B–E)** Representative electron micrographs of rod photoreceptor ribbon synapses in non-transduced 3 h light **(B)** and dark adapted **(C)**, and in AAV5-Pclo28-transduced 3 h light **(D)** and dark adapted **(E)** BALB/c mouse retina. Arrowheads point to plate-shaped synaptic ribbons, asterisks demarcate free floating or membrane attached ribbon spheres. **(F–I)** 3D-reconstructions of representative rod photoreceptor ribbon synapses from non-transduced 3 h light **(F)** and dark adapted **(G)**, and from AAV5-Pclo28-transduced 3 h light **(H)** and dark adapted **(I)** BALB/c mouse retina. Synaptic ribbon material is shown in black, ribbon-tethered vesicles in blue. bc: bipolar cell; hc: horizontal cell. Scale bar in **(E)** for **(B–E)** and in **(I)** for **(F–I)**: 0.5 μm.

The high percentage of membrane-attached spherical ribbon material in the adult AAV5-Pclo28-transduced rod photoreceptor terminals (Figure 6), suggests that Piccolino might be involved in the formation of the plate-shaped synaptic ribbons from precursor spheres. This hypothesis is supported by the fact that the synaptic spheres in the Piccolino knockdown photoreceptor terminals and the precursor spheres present during photoreceptor synaptogenesis are comparable in size with a mean diameter of ~130 nm (Regus-Leidig et al., 2009; this study, Table 1). Unfortunately, due to our experimental approach, we could not directly test whether Piccolino is involved in ribbon formation from precursor spheres. The Piccolo shRNA was injected between P5 and P7, and the majority of precursor spheres would have already matured into plate-shaped, anchored ribbons before the down regulation of Piccolino was effective. To circumvent this technical problem, we made use of the BALB/c mouse strain, which displays the dynamic process of ribbon disassembly and assembly in light and dark, respectively (Spiwoks-Becker et al., 2004; Fuchs et al., 2013). Our working hypothesis was that if Piccolino plays a role in the reassembly of synaptic ribbons from spherical ribbon material in BALB/c mouse photoreceptors in the dark, this process should be impaired in the Piccolino knockdown terminals. To test this hypothesis, we transduced BALB/c mouse retina with AAV5-Pclo28 and performed a detailed ultrastructural analysis, comparing non-transduced vs. transduced rod photoreceptors, after 3 h light or dark adaption (Figure 7). Importantly, measurements of Piccolino fluorescence intensities in the OPL of non-transduced BALB/c retinae did not differ between light and dark adaptation (light adapted: 1 ± 0.24 vs. dark adapted: 1 ± 0.28, normalized to the dark condition; *n* = 3 animals per adaptation state, 6 images per animal).

#### Non-transduced BALB/c retina, light and dark adapted

As expected, and in line with published data (Spiwoks-Becker et al., 2004; Fuchs et al., 2013), the highest percentage of the ribbon material in rod photoreceptor terminals from non-transduced retinae of light adapted BALB/c retina appeared as spheres in single ultrathin sections (56.3%; *n* = 314 ribbons; Figures 7A,B). 3D reconstructions of 10 rod photoreceptor synaptic sites revealed that the majority of the multiple spheres in a terminal were free floating (3.4 ± 1.1; mean ± *SD*), and only 1.5 ± 0.5 spheres (mean ± *SD*) were membrane attached (Figure 7F). In rod photoreceptor terminals from non-transduced areas of dark adapted BALB/c retina, most of the ribbon material appeared plate-shaped in single ultrathin sections (98.6%; *n* = 147 ribbons; Figures 7A,C), and horseshoe-shaped in 3D reconstructions (Figure 7G; *n* = 5 rod photoreceptor synaptic sites).

#### AAV5-Pclo28-transduced BALB/c retina, light and dark adapted

In rod photoreceptor terminals from light adapted AAV5-Pclo28-transduced BALB/c retina, the highest percentage of ribbon material appeared as spheres, a result comparable to the situation in non-transduced photoreceptor terminals. It is important to note, however, that different from the non-transduced terminals, the majority of spheres appeared membrane attached in single ultrathin sections (53.7%; *n* = 161 ribbons; Figures 7A,D). In rod photoreceptor terminals from dark-adapted AAV5-Pclo28-transduced BALB/c retina, the appearance of the ribbon material was strikingly different from that in non-transduced terminals. In line with our hypothesis that Piccolino might play a role in ribbon formation, only ~40% of the ribbon material appeared plate-shaped, and ~55% appeared as membrane attached spheres in single ultrathin sections (*n* = 159 ribbons; Figures 7A,E). 3D reconstructions confirmed the membrane-association of the spherical ribbon material in both light (Figure 7H; *n* = 6 rod photoreceptor synaptic sites) and dark adapted (Figure 7I; *n* = 5 rod photoreceptor synaptic sites) conditions.

Noteworthy is also the fact that in the Piccolino knockdown rod photoreceptor terminals of both C57BL/6 and BALB/c mouse retina, the mean diameter of the synaptic spheres, free floating as well as membrane attached, was comparable and resembled closely the mean diameter of ribbon precursor spheres present during photoreceptor synaptogenesis (Table 1).

## Discussion

A growing number of studies indicate that, despite a highly similar protein composition between the AZs of conventional chemical “brain” synapses and ribbon-type “sensory” synapses, a simple transfer of protein function from one AZ to the other is not feasible (Moser et al., 2006; Schmitz, 2009; Matthews and Fuchs, 2010; Regus-Leidig and Brandstätter, 2012). Most AZ proteins come in synapse specific isoforms and/or splice variants with specialized properties adapted to the individual needs in neurotransmission at different synapse types. In line with this, the continuously active retinal photoreceptor ribbon synapses contain a unique set of presynaptic proteins and protein variants, e.g., Syntaxin 3, RIBEYE, Complexins 3 and 4, and ubMunc13-2 (Morgans et al., 1996; Schmitz et al., 2000; Reim et al., 2005; Cooper et al., 2012). Recently, we showed that Piccolino, a splice variant of the CAZ protein Piccolo, is specifically present at ribbon-type synapses, and absent at conventional chemical synapses (Regus-Leidig et al., 2013).

To study the putative role of Piccolino at the photoreceptor ribbon synapse, we down regulated Piccolino in rod photoreceptors of the mouse retina via AAV-transduction *in vivo*, and examined with light and electron microscopy the impact of reduced Piccolino-levels on the structure of the presynaptic ribbon. In AAV5-Pclo28-transduced rod photoreceptor terminals from retinal areas with low transduction rates, synaptic ribbon ultrastructure was partially irregular and disrupted. The structural ribbon phenotype became most distinct in Piccolino knockdown photoreceptor terminals from retinal areas with high AAV5-Pclo28-transduction rates, leading to a marked loss of regular plate-shaped ribbons and an increase in spherical, membrane attached ribbon material (Figure 6). Spherical ribbon profiles in rod photoreceptor terminals have been described in a number of studies in the context of ribbon synapse disintegration and/or photoreceptor degeneration (Regus-Leidig et al., 2010b, 2014; Fuchs et al., 2012; Zabouri and Haverkamp, 2013). Importantly, however, in all these studies spherical ribbon profiles were shown to float freely in the photoreceptor terminals' cytoplasm. To our knowledge, a phenotype manifesting in predominantly plasma membrane-attached spherical ribbon material is unique and has not been observed before. Furthermore, it is important to stress that the size of the membrane attached spherical ribbon profiles in the Piccolino knockdown photoreceptor terminals is in accordance with the size of the precursor spheres found during photoreceptor ribbon synaptogenesis (Regus-Leidig et al., 2009). From these results we suggest that, different from a degenerative phenotype, the here described phenotype may reflect a structural defect in the assembly of the plate-shaped rod photoreceptor synaptic ribbon from precursor spheres, a process in which Piccolino seems to be involved. Unfortunately, we were not able to directly address this question, since ribbon formation precedes the expression of Piccolo shRNA in our experimental approach.

To change the time point of viral injection in our experiments was no option, as injections earlier than P5 led to severe retinal damage. In a second round of Piccolino knockdown experiments we therefore decided to switch from the C57BL/6 to the BALB/c mouse strain. BALB/c mice show a light dependent and reversible process of ribbon disassembly and assembly, a process not found in C57BL/6 mice (Spiwoks-Becker et al., 2004; Fuchs et al., 2013). Although the knockdown experiments in BALB/c mice are no direct test for the involvement of Piccolino in ribbon assembly, our results strongly support a role of Piccolino in the assembly of the plate-shaped synaptic ribbon from ribbon spheres: (1) in the light adapted AAV5-Pclo28-transduced rod photoreceptor terminals, the size of anchored spherical ribbon profiles was strikingly similar to the mean diameter of precursor spheres present during photoreceptor synaptogenesis (Regus-Leidig et al., 2009; this study), and (2) in the dark adapted AAV5-Pclo28-transduced rod photoreceptor terminals, the spherical ribbon profiles neither aggregated, nor adopted a plate-like shape (Figure 7).

The group of Frank Schmitz showed in an elegant study that heterologously expressed RIBEYE forms electron-dense aggregates that resemble spherical ribbon profiles (Magupalli et al., 2008). Plate-shaped ribbons were not observed in RIBEYE-transfected cells. The authors argued that spherical ribbon material represents the basal building unit of photoreceptor synaptic ribbons and additional ribbon components are needed to build a mature and fully functional plate-shaped ribbon from spherical ribbons (Magupalli et al., 2008). Based on our findings, we suggest that Piccolino is one of these components. However, there are some important questions left to be answered: Why are ribbons in auditory hair cells of the cochlea, which also contain Piccolino (Regus-Leidig et al., 2013), spherical and not plate-shaped? Is the proposed function of Piccolino universal for all types of ribbon synapses or is it valid only for photoreceptor ribbon synapses?

In addition to the disrupted ribbon morphology in rod photoreceptors, Piccolino knockdown also led to a slightly disorganized arciform density/plasma membrane compartment, as depicted by Bassoon and ubMunc13-2 staining (Figure 5). This suggests that ribbon formation and aggregation of arciform density/plasma membrane proteins may go hand in hand. In an earlier developmental study, we showed that arciform density/plasma membrane proteins like CAST1, Munc13, RIM2, and the L-type Ca^2+^ channel α1f subunit clustered at the AZ only sometime after the arrival of a first set of ribbon components (Regus-Leidig et al., 2009). Moreover, in Bassoon mutant photoreceptor terminals, in which synaptic ribbons are not anchored to the AZ, the arciform density looks similarly disorganized as seen in the present study (tom Dieck et al., 2005). More direct evidence for an organizing role of the synaptic ribbon in the assembly of the AZ comes from studies in zebrafish sensory hair cells, in which over-expression of RIBEYE was shown to cluster Ca_V_1.3a channels at ectopic sites (Sheets et al., 2011), and from mouse inner ear hair cells, in which ribbon absence reduced Ca^2+^ channel numbers in Bassoon mutant synapses (Frank et al., 2010). While there seems to be an interplay between the synaptic ribbon and arciform density proteins, the observation that the arciform density/AZ compartment is less disorganized than the synaptic ribbon in Piccolino knockdown terminals supports our hypothesis that Piccolino is involved in the structural organization of the synaptic ribbon.

**Figure 8:**
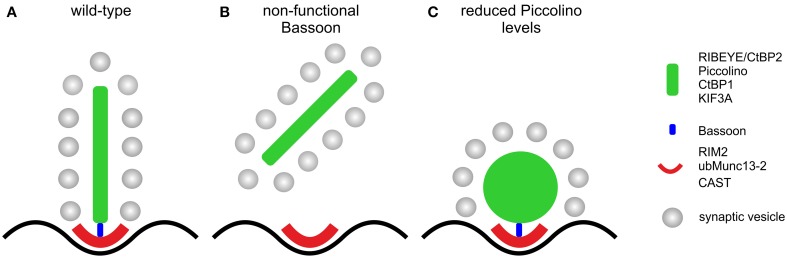
**Schematic illustration of the effect of the absence of Bassoon and Piccolino on the structure of the rod photoreceptor ribbon synaptic complex. (A)** Plate-shaped and anchored ribbon in a wild-type rod photoreceptor synapse. **(B)** Free floating ribbon in a Bassoon-mutant rod photoreceptor synapse. **(C)** Membrane attached spherical ribbon in a Piccolino knockdown photoreceptor synapse. Presynaptic proteins and their localization to either the ribbon (green) or the arciform density compartment (red) of the ribbon synaptic complex are depicted in the legend. Bassoon (blue) is the molecular link between the two compartments.

Our study provides evidence for the multifaceted role of the CAZ protein Piccolo in conventional and ribbon-type chemical synapses. In conventional chemical synapses, Piccolo and its homolog Bassoon seem to have greatly overlapping roles and can compensate each other's loss for at least some function (Altrock et al., 2003; Leal-Ortiz et al., 2008; Mukherjee et al., 2010; Waites et al., 2013). In photoreceptor ribbon synapses, the two CAZ proteins have adopted different functions in the different compartments of the presynaptic ribbon complex, resulting in the inability to compensate each other's loss (Dick et al., 2003; tom Dieck et al., 2005; Regus-Leidig et al., 2010a, 2013; this study). Whereas Bassoon is essential for ribbon anchoring during photoreceptor synaptogenesis (Dick et al., 2003), Piccolino seems to be involved in the formation of a plate-shaped ribbon (Figure 8). This division of labor at the rod photoreceptor ribbon synapse reflects the complex nature of and high functional demands on this sensory synapse. As structure suggests function, we finally propose that loss of a well-organized plate-shaped ribbon will compromise neurotransmission from rod photoreceptors, as was recently shown for the knockout of the AZ protein CAST in rod photoreceptors (tom Dieck et al., 2012). The knockout resulted in a reduction of the rod photoreceptor AZ size and caused diminished amplitudes of the b-wave in scotopic ERGs, which suggests that the rod photoreceptor release rate might scale with the size of the AZ. Initially we also performed multifocal ERG recordings from Piccolino knockdown animals (data not shown), and first results hinted at a reduced photoreceptor synaptic transmission in transduced retinal areas. We stopped with the recordings, however, as transduced retinal areas and multifocal ERG results varied far too much, making the results unreliable. Therefore, to study Piccolino's role in neurotransmission, we are in the process of generating a knockout mouse in which all known Piccolo variants are deleted.

## Author contributions

Hanna Regus-Leidig, Johann H. Brandstätter designed research, Hanna Regus-Leidig, Michaela Fuchs, Martina Löhner, Sarah R. Leist, Sergio Leal-Ortiz, Vince A. Chiodo conducted research, Hanna Regus-Leidig, Michaela Fuchs, Martina Löhner, analyzed data, William W. Hauswirth, Craig C. Garner supplied reagents, Hanna Regus-Leidig, Johann H. Brandstätter wrote the paper.

### Conflict of interest statement

The authors declare that the research was conducted in the absence of any commercial or financial relationships that could be construed as a potential conflict of interest.
